# Synthesis and Characterization of Bipyridine-Based Polyaminal Network for CO_2_ Capture

**DOI:** 10.3390/polym14183746

**Published:** 2022-09-07

**Authors:** Nazeeha S. Alkayal, Maha M. Alotaibi, Nada Y. Tashkandi, Maymounah A. Alrayyani

**Affiliations:** Chemistry Department, Faculty of Science, King Abdulaziz University, P.O. Box 80203, Jeddah 21589, Saudi Arabia

**Keywords:** polyaminal-linked polymers, melamine, CO_2_ uptake, Bipyridine

## Abstract

The response to the high demand for decreasing the amount of CO_2_ in the atmosphere, a new polyaminal-based polymer network was designed and successfully prepared through one-pot polycondensation reaction of melamine and [2,2′-Bipyridine]-5,5′-dicarbaldehyde. The formation of the polymer structure was confirmed by FT-IR, solid-state ^13^C NMR, and powder-X-ray diffraction. The porous properties of the polymeric structure were confirmed by field-emission scanning electron microscope and N_2_ adsorption–desorption methods at 77 K. The prepared polymer can take up 1.02 mmol/g and 0.71 mmol/g CO_2_ at 273 K and 298 K, respectively, despite its relatively modest Brunauer–Emmett–Teller (BET) surface area (160.7 m^2^/g), due to the existence of superabundant polar groups on the pore surfaces.

## 1. Introduction

Organic-based polymers such as porous organic polymers (POPs), covalent organic frameworks (COFs), and metal-organic frameworks (MOFs) are an important class of materials that paved the way for many applications such as carbon dioxide capture, hydrogen storage, adsorption of volatile organic compounds, heterogeneous catalysis, chemical sensing, materials for batteries, light harvesting, and photoluminescence [1,2,3,4,5,6,7].

On the other hand, POPs have received far more attention from scientists than other polymers owing to their efficiency in a variety of applications as well as their ease of synthesis via one-pot condensation. Furthermore, POPs can be functionalized with various moieties in order to tune their properties and enhance their efficiency [1,2,3]. 

Global warming mainly occurs when CO_2_ concentration increases abundantly in the atmosphere. Scientists have long been dedicated to overcoming this issue by preparing sorbent materials to decrease the concentration of CO_2_ [4,5]. In this regard, the polyaminal-based POPs provide an efficient platform for CO_2_ capture and enhance the porosity parameters owing to their rich nitrogen system and facile synthesis pathways [7,8,9,10,11]. Exploiting the benefit of the formation of a stable aminal-linked network, hence polyaminal adsorbents have been extensively prepared through one-pot catalyst-free polycondensation between aldehyde moieties and melamine [12,13,14,15,16,17]. Meng Rong and co-workers successfully synthesized three triphenylamine-based polyaminal networks (TMPs) by polycondensation of triphenylamine-based aldehydes with melamine. They explored that presence of more formyl groups would increase the surface area and provide high porous materials [18].

Furthermore, the introduction of a polycyclic aromatic compound in the polymeric structure could also increase the porosity and the efficiency of CO_2_ capture owing to their rich electron conjugated systems. Accordingly, naphthalene-based polyaminal networks (PAN-NA) were designed and explored their properties [18,19,20]. Alkayal et al. confirmed that the effect of the introduction of α-naphthaldehyde to improve the efficiency of CO_2_ capture of PAN-NA was clearly noticed; uptake increased to 133 mg/g at 273 K and one bar. Furthermore, the adsorption behavior of PAN-NA for different metal cations such as Pb(II), Cr(III), Cu(II), Cd(II), Ni(II), and Ba(II) was explored; an excellent adsorption selectivity toward the Pb(II) cation was pronounced [21]. 

Inspired by the previous work [21], herein, we report the fabrication of porous organic polymer based on melamine and [2,2′-Bipyridine]-5,5′-dicarbaldehyde via a one-pot condensation approach. The prepared polymer (Bipy-PAN) was characterized by utilizing FT-IR, solid-state 13C NMR, powder-XRD, and SEM analysis. Porosity was examined using surface (BET). The gases (CO_2_ and N_2_) uptakes are performed by the prepared polymer

## 2. Experimental Section 

### 2.1. Materials 

All experiments were carried out under an atmosphere of argon. [2,2′-Bipyridine]-5,5′-dicarbaldehyde was purchased from ShanghaiSunchemlnc, and melamine (97.5%) and Dimethyl sulfoxide (DMSO 99%) were purchased from BDH laboratory reagents. Tetrahydrofuran (THF ≥ 99.5%) and dichloromethane (≥99.8%) were purchased from Fisher chemicals, as well as acetone (99.5%). All other materials were purchased from commercial sources and used without further purification.

### 2.2. Synthesis of Porous Organic Polymers Based on Melamine and [2,2′-Bipyridine]-5,5′-Dicarbaldehyde

A dry three-necked flask equipped with a stirrer and a condenser was degassed using one evacuation-argon-backfill cycles. The flask was evacuated first using vacuum. Under argon flow, melamine (0.5 g, 3.96 mmol), [2,2′-Bipyridine]-5,5′-dicarbaldehyde (0.7 g, 5.94 mmol) and 30 mL DMSO were added and heated at 175 °C for 72 h. The system was cooled down, and the solid was isolated and washed successively with 30 mL of dimethyl formamide, dichloromethane, and acetone. The resulting white solid was dried in a vacuum at 60 °C for 3 h to afford a yield of 85%. 

### 2.3. Instrumentation

NMR spectra were recorded on a Varian Inova 400 or Inova 600 MHz NMR spectrometer using C_6_D_6_ or THF-*d*_8_ as the solvent. The NMR standards used are as follows: ^1^H NMR spectra were referenced to residual C_6_D_5_H (7.15 ppm) or THF-*d_7_*H (3.58 ppm); ^13^C NMR spectra were referenced to the central transition of C_6_D_6_ (128.00 ppm). FT-IR spectra were recorded as thin films using a Bruker Tensor 27 Spectrometer (resolution of 4 cm^−1^). Thermogravimetric analysis (TGA) was carried out on a TG-DTA6300 (Shimadzu, Kyoto, Japan) with a 10 °C min^−1^ heating rate in an interval of 25–500 °C under N_2_ atmosphere. X-ray diffraction (XRD) patterns were studied using a Bruker D8 Advance with Cu Kα radiation (wavelength 1.5418 Å) at 40 kV and 40 mA. The patterns were collected between 2θ of 10° and 60°, and the scan speed was 1.5 degree/min. N_2_ adsorption–desorption measurements were conducted on a Micromeritics 3 Flex 3500. The Brunauer–Emmett–Teller (BET) and Langmuir methods were conducted to measure the surface area of the material. The t-plot was used to approximate the micropore surface area. Before analysis, the sample was degassed by heating at 120 °C for 12 h under vacuum.

## 3. Result and Discussion 

### Synthesis and Characterisation

**Scheme 1:**
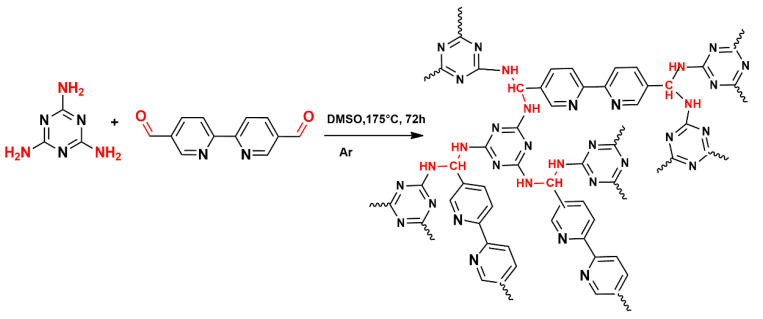
Synthetic route for the preparation of bipyridine-based polyaminal network (Bipy-PAN).

The polyaminal polymer was synthesized using a one-step polycondensation reaction of melamine with 2,2′-Bipyridine]-5,5′-dicarbaldehyde at 175 °C in an inert atmosphere as depicted in Scheme 1. 

**Figure 1:**
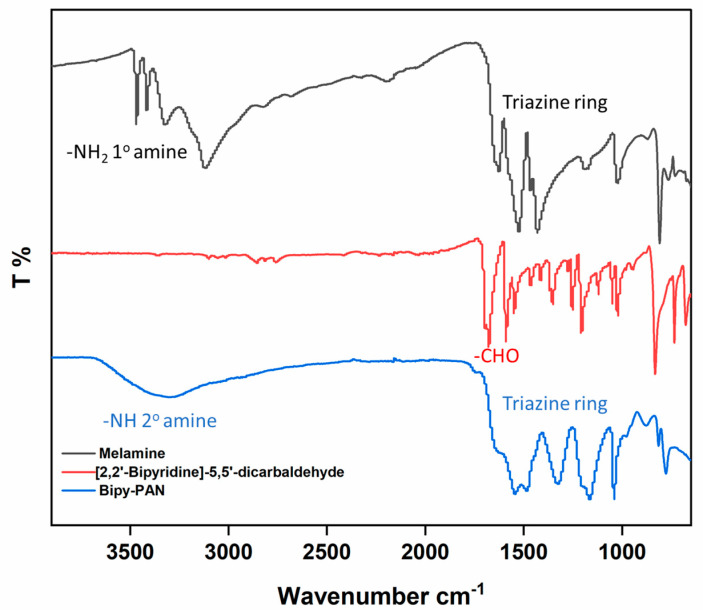
FT-IR spectra of melamine, pyridine dialdehyde and BiPy-PAN.

The successful formation of a cross-linked polyaminal network was confirmed using the FT-IR spectrum (indicated in the blue line in Figure 1.). The broad adsorption band at 3300 cm^−1^ in the FT-IR spectrum was assigned to the secondary amine (N–H) of the aminal moiety. Stretching vibrations of pure melamine (indicated in black) at 3470, 3420, and 1650 cm^−1^ for –NH2 stretching and –NH2 in the melamine unit, disappeared upon the reaction of [2,2′-Bipyridine]-5,5′-dicarbaldehyde with melamine forming Bipy-PAN. Stretching vibrations of pure [2,2′-Bipyridine]-5,5′-dicarbaldehyde (indicated in red) at 1610 cm^−1^ assigned to the CHO disappeared upon the formation of Bipy-PAN. The aminal moiety is also confirmed by the characteristic bands at 1340 cm^−1^ assigned to the (CN) group. The characteristic adsorptions of triazine rings are observed at 1510 and 1490 cm^−1^ [21], which confirms the existence of a melamine ring in the polymer. Moreover, the lack of azomethine C=N stretching resonance around 1600 cm^−1^ confirms the formation of an aminal bond in the provided reaction condition. 

**Figure 2:**
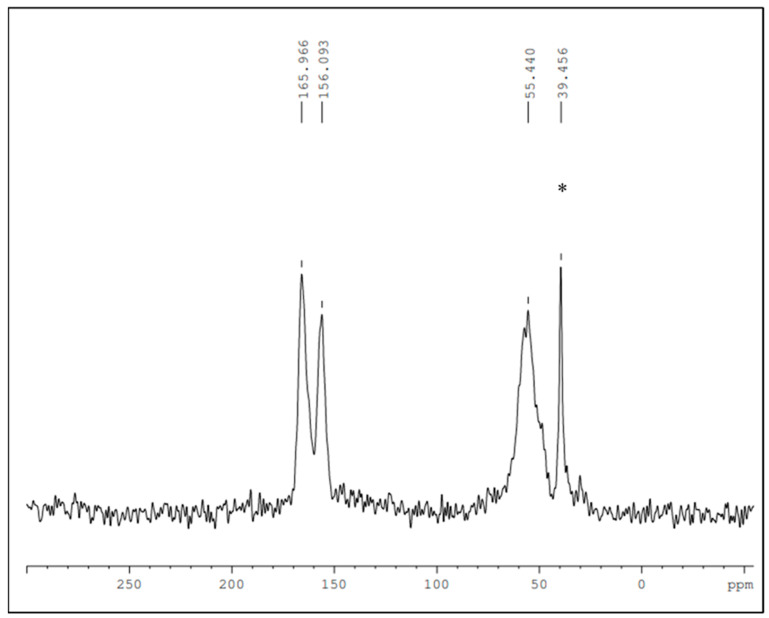
^13^C NMR spectrum of BiPy-PAN (*) peak arising from spinning sidebands.

To further confirm the structures, the solid-state ^13^C CP/MAS NMR measurements were recorded (Figure 2). The strong signal at 165.9 ppm was assigned to carbons in the triazine rings. A signal at 156 ppm was assigned for the triazine ring carbons of the second ring, as each methylene group is attached to two melamine rings, and each one experiences a different environment depending on cross-linking. The broad signal at 55.4 ppm was assigned to the methylene group in aminal linkages. Our results agree with the observed signal for the PAN-NA polymer, where signals of triazine ring were observed at 166 ppm, and a signal at 53.9 ppm was assigned to the methylene group in aminal linkages [21].

**Figure 3:**
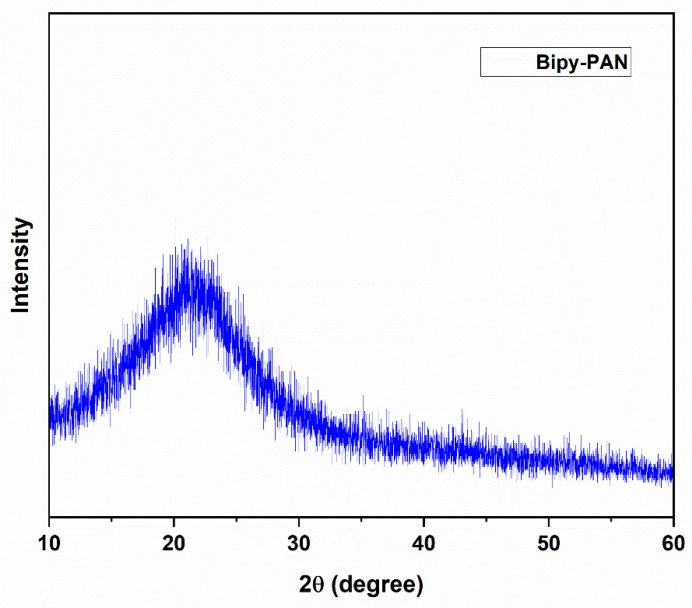
XRD pattern of Bipy-PAN.

For structural investigation, Figure 3 and Figure S1 show the XRD signals of BiPy-PAN and for the starting materials, melamine and [2,2′-Bipyridine]-5,5′-dicarbaldehyde, respectively. The crystallinity of the starting materials are revealed by the unique diffraction peaks in Figure S1, where melamine has scattering angles of 2θ = 13°, 15°, 22°, 26°, 27°, 28.9°, 30°, 34.8°, 35.9°, and 39° and [2,2′-Bipyridine]-5,5′-dicarbaldehyde has diffraction peaks at 2θ = 5°, 15°, 17°, 17.5°, 19°, 22°, 24°, 25°, 27.5°, and 32.3°. All sharp peaks can be regarded to be high crystallinity nature. Figure 3 displays only broad and wide diffraction peaks located at 2θ of 20° are found for BiPy-PAN, suggesting to their amorphous nature. The lack of any sharp peaks in Figure 3 indicates that all starting materials have been reacted.

**Figure 4:**
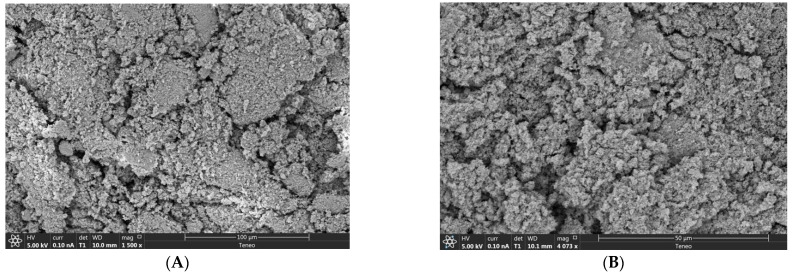
SEM images of Bipy-PAN, scale bar = 100 µ (**A**), 50 µ (**B**).

The surface morphology of the polyaminal polymer (Bipy-PAN) was investigated using SEM. Figure 4 shows agglomerated particles (cotton-like structures) as observed previously for some of the polyaminal polymers [7,22]. 

**Figure 5:**
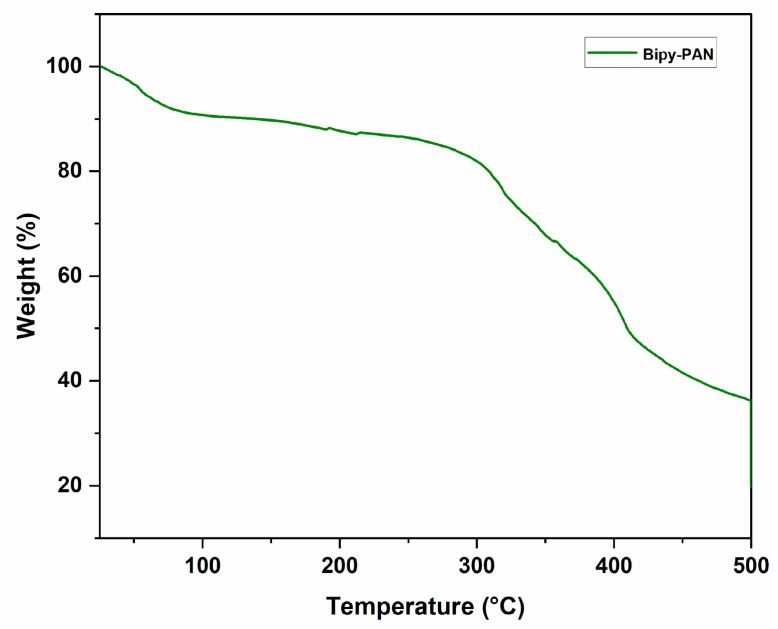
TGA curve of Bipy-PAN and air flow (10 °C/min).

The thermal stability of Bipy-PAN material was investigated by TGA analysis, and the obtained Thermogram is presented in Figure 5. The first weight loss (9%) was around 125 °C, which is assigned to the weight loss of the solvent. The main weight loss started at a temperature over 355 °C due to the breakdown of the network, and the burning began gradually above 410 °C. Therefore, Bipy-PAN is more stable up to 355 °C.

**Figure 6:**
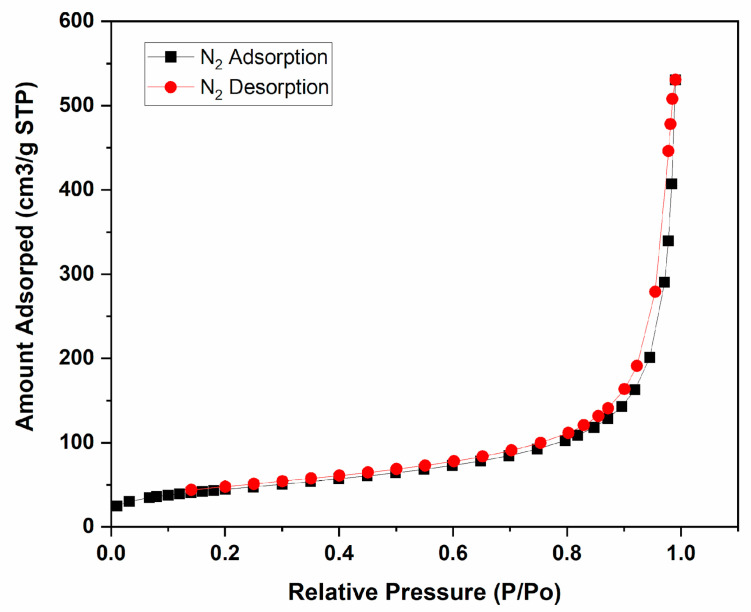
N_2_ adsorption–desorption isotherms of Bipy-PAN measured at 77 K.

Nitrogen sorption studies at 77 K (Figure 6) exhibit a typical type IV isotherm with a small H4 hysteresis loop, implying a solely mesoporous structure. The Brunauer–Emmett–Teller (BET) surface area is evaluated to be 160.7076 m^2^/g (Figure S2), and the t-plot external surface area is 153.2645 m^2^/g. The small difference between BET and t-plot surface areas of Bipy-PAN suggests that these specimens almost have 7.4431 m^2^/g microporous channels. The pore size of Bipy-PAN calculated by the Barrett–Joyner–Halenda method (BJH) shows that Bipy-PAN has major peaks centered at ~53 nm with a significantly broader distribution (Figure S3), and the total pore volume of pores less than 37 nm at p/p° = 0.94 is 0.31 cm^3^/g.

**Figure 7:**
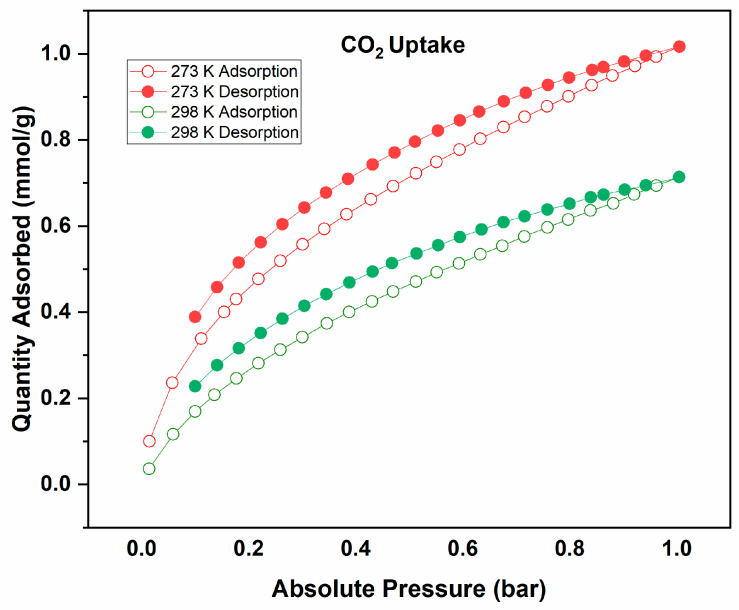
CO_2_ uptake isotherms of Bipy-PAN obtained at 273 and 298 K.

The CO_2_ adsorption performance of Bipy-PAN is assessed by CO_2_ isotherms at 273 K and 298 K, where both are measured up to one bar (Figure 7). It is observed that the CO_2_ uptakes of Bipy-PAN are ranged 0.1002–1.016 mmol/g at 273 K and 0.0363–0.7132 mmol/g at 298 K. Remarkably, the CO_2_ uptake of Bipy-PAN at 273 K is much higher than that at 298 K, implying an exothermic physical adsorption process. The great affinity to capture CO_2_ in both temperatures is attributed to the relatively high BET surface area and the abundance of nitrogen in the Bipy-PAN skeleton. It is noticeable that the CO_2_ adsorption of Bipy-PAN is comparable to or relatively superior to some other melamine-based POPs reported in the literature (Table 1) [23,24,25]. Since the flue gas contains 3–15% CO_2_ at a total pressure of ~1 bar, the CO_2_ adsorption of Bipy-PAN at ~0.15 bar is more relevant to practical CO_2_ uptake [26]. At 0.15 bar and 273 K, Bipy-PAN adsorbs ~0.4002 mmol/g CO_2_, remaining higher than that at the same low pressure and 298 K (0.2462 mmol/g).

## 4. Conclusions

A new polyaminal-based porous polymeric network from melamine and 2,2′-Bipyridine-5,5′-dicarbaldehyde was synthesized by one-pot polycondensation. The structure and porous properties of the novel prepared polymer Bipy-PAN were investigated. The gas sorption experiments indicate that the synthesized polymer has a high CO_2_ storage capacity due to the presence of numerous polar groups distributed through its network. Bipy-PAN, with a Brunauer–Emmett–Teller (BET) surface area of about 160.7 m^2^/g, can uptake 1.02 mmol/g and 0.71 mmol/g CO_2_ at 273 K and 298 K, respectively, which makes this polymer a promising gas adsorbent candidate for environmental applications, such as gas storage or separation and catalysis.

## Figures and Tables

**Table 1:** The *S*_BET_ surface area and the CO_2_ uptake of various melamine-based POPs.

	*S*_BET_ (m^2^/g)	CO_2_ Uptake (mmol/g)		Ref.
		273 K	298 K	
PA-1	84.5	0.134	0.09	[24]
PA-2	22	0.086	0.054	[24]
PTPA-0.5	157	0.53	0.32	[25]
PTPA-1	230	0.96	0.67	[25]
PTPA-2	215	1.03	0.73	[25]
Bipy-PAN	160.7	1.02	0.71	This work

## Data Availability

Not applicable.

## Supplementary Materials

The following supporting information can be downloaded at: https://www.mdpi.com/article/10.3390/polym14183746/s1. Figure S1. XRD pattern of [2,2’-Bipyridine]-5,5’-dicarbaldehyde and melamine. Figure S2. BET plots of Bipy-PAN obtained from N_2_ isotherms at 77 K. Figure S3. Pore size distribution in Bipy-PAN, as determined by BJH analysis.
