# Supplementary material for: Synthesis and Characterization of Bipyridine-Based Polyaminal Network for CO2 Capture
Source: Polymers (Basel). 2022 Sep 7;14(18):3746. doi: 10.3390/polym14183746 (PMC9502079; doi:10.3390/polym14183746)
Supplement: Supplementary file 1 [file polymers-14-03746-s001.zip › polymers-1894169-supplementary.pdf]

polymers

MDPI

Supplementary Materials: Synthesis and Characterization of Bipyridine-Based Polyaminal Network for CO_2 Capture

Nazeeha S. Alkayal, Maha M. Alotaibi, Nada Y. Tashkandi and Maymounah A. Alrayyani

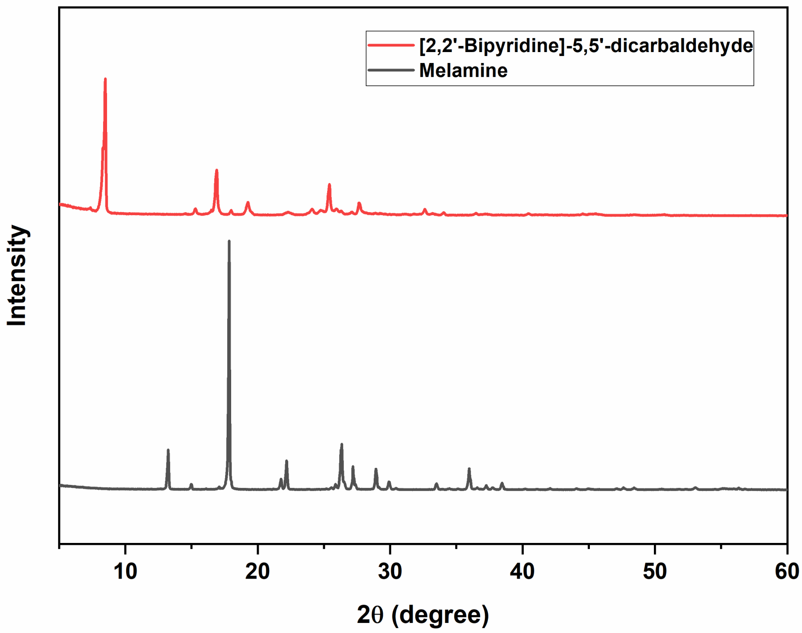

Figure S1. XRD pattern of [2,2'-Bipyridine]-5,5'-dicarbaldehyde and melamine.

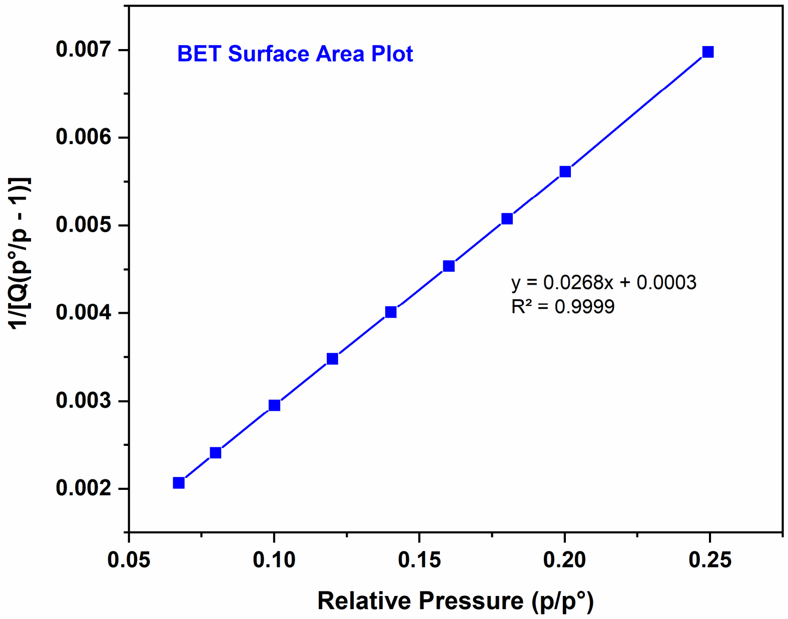

Figure S2. BET plots of Bipy-PAN obtained from N_2 isotherms at 77 K.

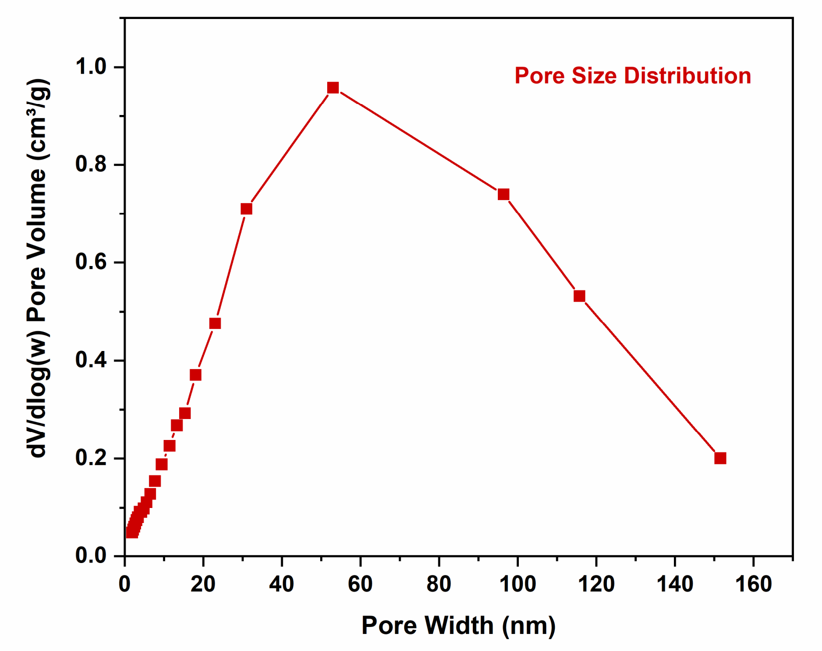

Figure S3. Pore size distribution in Bipy-PAN, as determined by BJH analysis.
